# Optimizing time, cost, and carbon in construction: grasshopper algorithm empowered with tournament selection and opposition-based learning

**DOI:** 10.1038/s41598-023-49667-0

**Published:** 2023-12-14

**Authors:** Vu Hong Son Pham, Phuoc Vo Duy, Nghiep Trinh Nguyen Dang

**Affiliations:** Faculty of Civil Engineering, Ho Chi Minh City University of Technology (HCMUT), Vietnam National University (VNU-HCM), Ho Chi Minh City, Vietnam

**Keywords:** Civil engineering, Computational science

## Abstract

The global construction industry plays a pivotal role, yet its unique characteristics pose distinctive challenges. Each construction project, marked by its individuality, substantial value, intricate scale, and constrained adaptability, confronts crucial limitations concerning time and cost. Despite contributing significantly to environmental concerns throughout construction activities and infrastructure operations, environmental considerations remain insufficiently addressed by project managers. This research introduces an improved rendition of the muti-objective grasshopper optimization algorithm (MOGOA), termed eMOGOA, as a novel methodology to tackle time, cost, and carbon dioxide emission trade-off problems (TCCP) in construction project management. To gauge its efficacy, a case study involving 29 activities is employed. eMOGOA amalgamates MOGOA, tournament selection (TS), and opposition-based learning (OBL) techniques to enhance the performance of the original MOGOA. The outcomes demonstrate that eMOGOA surpasses other optimization algorithms, such as MODA, MOSMA, MOALO and MOGOA when applied to TCCP. These findings underscore the efficiency and relevance of the eMOGOA algorithm within the realm of construction project management.

## Introduction

The construction industry holds a position of paramount importance on the global stage; however, it grapples with distinctive challenges stemming from its inherent nature. Each construction project possesses inherent uniqueness, substantial value, intricate scale, and limited modifiability. In the course of operational phases, conflicts frequently arise among the involved stakeholders. Time, cost, and quality stand as pivotal constraints within construction undertakings. Consequently, it is not only quality that bears significance but also the factors of time and cost that assume pivotal roles in attaining success in a construction project. The optimization of cost and time emerges as a central and formidable undertaking within the panorama of construction project management. Navigating the balance between time and cost presents a recurring challenge for project managers as they strive to accomplish projects within stipulated timelines while simultaneously minimizing expenditures^1^. The optimization of cost and time within construction management confers a multitude of advantages for construction enterprises, encompassing the preservation of resources, assurance of punctual delivery, and the cultivation of avenues for business growth^2^.

The construction industry assumes a pivotal role in contributing to a diverse array of environmental concerns, encompassing both the processes of construction activities and the subsequent operation of structures^3,4^. These processes wield a central influence on outdoor environmental pollution and the release of greenhouse gas emissions. Remarkably, the production of construction materials is attributed to the highest levels of carbon dioxide (CO_2_) emissions. Despite critical benchmarks such as time, cost, and quality that define success in the majority of construction projects, environmental considerations rarely receive commensurate attention from project managers. Presently, a scarcity of research is evident within the domain of optimizing cost and progress while concurrently addressing environmental issues. A subset of studies has undertaken the formulation of models that engage in a trade-off between construction costs and carbon dioxide emissions. These studies adopt multi-objective optimization approaches grounded in combinatorial algorithms^5^.

A significant majority of prior research endeavours have predominantly focused on the utilization of a fundamental relationship known as "Finish to Start" (FS). According to this relationship, an activity can only commence subsequent to the completion of its preceding activities. However, this stringent principle may not seamlessly align with the intricate realities inherent in actual projects. In practical project management, professionals frequently optimize project schedules by orchestrating the execution of concurrent activities within the project network^6,7^. For instance, in the context of high-rise building construction projects, tasks such as wall construction and painting can be carried out concurrently, facilitated by the incorporation of distinct initiation times for each of these activities. This pragmatic approach diverges from a strictly linear arrangement of tasks, embracing a more malleable and adaptive scheduling methodology.

Within the framework of an activity-on-node diagram, a construction project takes form, delineating an intricate network of *m* nodes and the interlinkages that establish relationships between activities. Each discrete activity embedded in this framework presents a range of execution alternatives, each inherently tied to predefined factors such as time, cost, and CO2 emissions. Central to the optimization effort of the TCCP framework is the pursuit of tripartite minimization: reducing project duration, managing costs, and mitigating carbon dioxide emissions. This multifaceted objective is achieved through the careful selection of the most suitable execution approach for each activity.

The primary objective revolves around the reduction of project duration, denoted as *Tp*, and is elucidated by the following equation:

1 $${T}_{p}={\text{min}}(\underset{z=1,\dots , Z}{{\text{max}}}({\mathit{TS}}_{z}+{D}_{z}))={\text{min}}(\underset{z=1,\dots ,Z}{{\text{max}}}({TF}_{z}))$$

$Tp\,\mathrm\,{ is \,the \,project \,duration};$ *TS*_*z*_ is the start time of activity *z*; *TF*_*z*_ is the completion time activity *z*; *D*_*z*_ is the duration of activity *z.*

The subsequent objective involves the reduction of the overall project cost, described by the following equation:

2 $${C}_{P}={C}_{D}+{D}_{u}=\sum_{z=1}^{Z}{c}_{z}+{C}_{o}+b\times {T}_{P}$$

$Cp\,\mathrm\,{ is\, the\, overall\, project\, cost};$ *C*_*D*_ is the sum of individual direct cost; *C*_*o*_ is the financial expenses; $b\times {T}_{P}$ is the cost corresponding to the cumulative project duration.

The last objective centers on minimizing the total carbon dioxide emissions from the project, detailed in the following equation:

3 $$C{E}_{P}=\sum_{z=1}^{Z}{\left({e}_{d}+{e}_{in}\right)}_{z}$$

*CE*_*p*_ is the total carbon dioxide emissions; *e*_*d*_ is the direct carbon dioxide emissions; *e*_*in*_ is the indirect carbon dioxide emissions.

4 $${e}_{d}={Q}_{ed}\times {F}_{e}+{Q}_{dd}\times {F}_{d}$$

*Q*_*ed*_ is the electricity consumption; *Q*_*dd*_ is the diesel consumption; *F*_*e*_ is the carbon emission factor (CEF) attributed to each unit of electricity consumption; *F*_*d*_ is the CEF for each unit of diesel consumption.

5 $${e}_{in}=\sum_{l=1}^{n}({Q}_{l}\times {F}_{l}+{Q}_{el}\times {F}_{e}+{Q}_{dl}\times {F}_{d})$$

*Q*_*l*_ is the usage of material *l* within the activity; *Q*_*e*_ is the electricity consumption related to the transportation of material *l* for the activity; *Q*_*d*_ is the diesel consumption pertaining to the transportation of material *l* for the activity; *F*_*l*_ is the *CEF* for each unit of material *l* production.

The innovation and primary objectives of this research are delineated as follows:

- Implementation of an enhanced version of the MOGOA in the context of TCCP
- Incorporation of diverse relationship types in project scheduling, such as "start to finish" and "finish to finish."
- Development of a three-dimensional Pareto front as a decision-making tool.

The forthcoming section will provide a comprehensive review of the prevailing literature concerning TCCP, tournament selection, and opposition-based learning. “Optimization model development” will delve into the intricate process of formulating the proposed model, while "Computational experiments" will offer an exhaustive analysis of the validation results, thereby effectively showcasing the efficacy and performance of the model. Finally, "Discussion" will encapsulate the study's conclusions and delineate potential pathways for future research.

## Literature review

### Grasshopper optimization algorithm

Amidst the rise of machine learning^8^, the academic world has also shown a growing interest in optimization algorithms, acknowledging their potent capabilities in tackling intricate optimization problems. The grasshopper optimization algorithm (GOA)^9^ draws inspiration from the swarming behaviour of grasshoppers in nature. It's an optimization method meticulously crafted for a wide range of applications, spanning engineering, science, economics, and other disciplines. By leveraging the core principles of grasshopper swarming, GOA effectively explores and identifies optimal solutions. Wu et al.^10^ presented a trajectory optimization method tailored for solar-powered UAVs in urban settings. Their approach utilized an adaptive version of GOA within a distributed model predictive control framework, addressing specific challenges like sight occlusions. This method enhances both precision and real-time applicability. Barman et al.^11^ proposed a region-specific short-term load forecasting model for Assam, India. Their model amalgamated GOA with support vector machines (SVM), considering the unique climatic factors of the region. Notably, their approach surpassed traditional models and other hybrid techniques, such as GA-SVM and PSO-SVM, in accuracy.

El‐Fergany^12^ applied GOA to ascertain optimal values for seven parameters of proton exchange membrane fuel cells (PEMFCs) stack models. The objective was to minimize discrepancies between experimental and predicted outcomes. Wang et al.^13^ introduced the SACLMOGOA, an augmented multi-objective GOA. This algorithm aims to overcome challenges such as slow convergence and susceptibility to local optima inherent in the traditional MOGOA. The efficacy of this method was demonstrated in optimizing urban rail hybrid energy storage systems, specifically for the Changsha Metro Line 1 in China. Bukar et al.^14^ proposed an optimized rule-based energy management system (EMS) employing GOA for capacity planning in off-grid microgrids. Their approach manifested significant improvements, including reduced fuel consumption, CO_2_ emissions, and energy costs in comparison to conventional methods. Darvish Falehi^15^ presented a robust disturbance observer-based sliding mode controller (RDO-SMC) to function as a power system stabilizer (PSS). This controller aims to enhance dynamic stability by managing power system low-frequency oscillations via non-linear control of the excitation system, with optimization achieved using the multi-objective GOA. However, when faced with complex optimization tasks marked by multidimensionality and multimodality, GOA can experience convergence issues and may sometimes tend toward local optima^16^.

### Time, cost, and carbon dioxide emission trade-off problems (TCCP)

Numerous stochastic optimization methodologies have been implemented within the realm of construction project management to reconcile the competing objectives of time and cost limitations (as highlighted in Table 1). Several algorithms have drawn inspiration from behavioral patterns observed in wildlife native to their ecosystems. Aminbakhsh and Sonmez^17^ presented a particle swarm optimization (PSO) model tailored for medium and large-scale construction projects, showcasing enhanced performance in time–cost optimization problems (TCP) compared to prevailing methods. Son and Nguyen Dang^18^ introduced the multi-verse optimizer as a promising stochastic optimization approach for TCP, demonstrating its superiority over other techniques particularly in small-scale scenarios. Parveen and Saha^19^ proposed a multi-objective methodology employing genetic algorithms, incorporating a modified adaptive weight approach based on time and cost inputs. This facilitates the determination of optimal solutions and Pareto fronts to enable informed decision-making. Son and Nguyen Dang^20^ devised a hybrid multi-verse optimizer model combining the multi-verse optimizer and sine cosine algorithm. This innovative approach effectively addresses TCP in construction project management, yielding superior solutions for large-scale and intricate projects compared to preceding algorithms.

**Table 1:** Survey on related works.

References	Problem	Objective	Algorithms	Case study
Aminbakhsh and Sonmez^17^	TCP	Multi	PSO	Medium and large-scale construction projects
Son and Nguyen Dang^18^	TCP	Multi	MVO	Small-scale construction projects
Parveen and Saha^19^	TCP	Multi	GA	Small-scale construction projects
Son and Nguyen Dang^20^	TCP	Multi	hDMVO	Medium and large-scale construction projects
Liu, Meng^22^	TCCP	Multi	Refined PSO	Small-scale construction projects
Huynh, Nguyen^25^	TCQCP	Multi	MOSGO	Small and medium-scale construction projects
Lotfi, Yadegari^56^	RRCTCQEPTP	Multi	GAMS Augmented ε-constraint	Bridge construction projects
Lotfi, Kargar^57^	RCTCQEETPBCTRR	Multi	RRCTCQEPTP	Health care project
Lotfi, Haqiqat^58^	3RBAP	Single	MILP	Health care project
Lotfi, Nayeri^59^	Determining the start times and ordering plans	Single	GA	Two-period projects

*RRCTCQEPTP* robust resource constraint time–cost–quality–energy–pollution trade-off problem, *RCTCQEETPBCTRR* resource-constrained time–cost–quality–energy–environment tradeoff problem by considering BCT, risk and robustness, *3RBAP* robust, resilience, and risk-averse budget allocation for projects, *MILP* mix-integer linear programming.

In recent years, project managers have expanded their purview to encompass crucial determinants that extend beyond temporal and financial considerations, exerting a substantial impact on project success. Gupta and Trivedi^21^ introduced the AEHO model, a fusion of Apriori-based swarm intelligence and elephant herding optimization, proficiently optimizing construction time, cost, and environmental factors. This model outperforms the prevailing PSO model in terms of performance. Liu et al.^22^ proposed a BIM-oriented design optimization method that utilizes a refined PSO algorithm to enhance sustainability by balancing life cycle costs and carbon emissions. Yi et al.^23^ presented the Stochastic Carbon Emission Estimation method, which addresses the reduction of greenhouse gases by optimizing resource combinations through micro-scale operational modeling while adhering to time and cost constraints. He et al.^24^ introduced an innovative trilateral optimization model for high-rise construction, employing construction process and principal component analyses to facilitate optimal activity selection. Huynh et al.^25^ put forward MOSGO, an efficient approach for optimizing time, cost, quality, and carbon emissions (TCQCP) in construction. This approach demonstrates superiority over alternative algorithms. Sharma and Trivedi^26^ introduced the RMOSM, utilizing a discrete opposition-based genetic algorithm for robust multi-objective scheduling in large-scale projects. This method proves effective in addressing intricate trade-off problems.

### Tournament selection (TS)

The tournament selection (TS) mechanism, a fundamental approach within evolutionary algorithms, entails randomly sampling individuals from a population to engage in competitive contests. Individuals demonstrating superior fitness within each tournament are subsequently selected as parents for the next generation, thus striking a delicate balance between exploiting favorable solutions and exploring the solution space. Shehab et al.^27^ enhanced the Moth Flame Optimization (MFO) algorithm by combining it with hill climbing (HC) and the TS method. This synthesis demonstrates the superiority of the amalgamation over alternative iterations and algorithms across diverse evaluations. Manoharan and Boggavarapu^28^ introduced an innovative framework for hyperspectral image classification, integrating a customized version that fuses the whale optimization algorithm and the TS approach. This integration proves effective in yielding improved classification outcomes. Bakhshaei et al.^29^ proposed a novel optimization strategy, CSATSdiff, designed to determine optimal power exchange and incentive rates in grid-connected photovoltaic/pumped hydro storage systems. This approach leads to reduced operational expenditures. Zhenxing et al.^30^ presented the chaos antlion optimization algorithm, crafted to address issues of premature convergence and localized optima. The algorithm employs chaos-based population initialization, dynamically modulated parameters, tournament-driven antlion selection, and logistic chaos operators for ant optimization. This approach demonstrates accelerated convergence and heightened accuracy when tackling complex, high-dimensional benchmark functions and path planning problems, contrasting with standard antlion and other optimization methodologies. Introducing the tournament-based harmony search (THS) algorithm, Al-Betar et al.^31^ addressed the economic load dispatch (ELD) challenge by enhancing the convergence attributes of the harmony search (HS) algorithm through the incorporation of TS. This enhancement significantly improves solution quality compared to existing methodologies, across various test systems and scales.

### Opposition based learning (OBL)

Opposition-based learning (OBL) introduces an innovative paradigm by capitalizing on the insight that juxtaposing or providing "opposite" solutions can yield valuable perspectives within optimization and problem-solving endeavors. This approach, which aims to explore both optimal solutions and their contrasting counterparts, strives to enhance the efficiency and effectiveness of various computational algorithms^32^. Wang et al.^33^ presented GOPSO, an enhanced PSO algorithm that integrates generalized opposition-based learning (GOBL) and Cauchy mutation to mitigate premature convergence. This enhancement results in improved performance across diverse benchmark functions. Shaw et al.^34^ introduced OGSA, a fortified gravitational search algorithm that employs OBL for initial population setup and generation transitions, subsequently leading to enhanced convergence rates and competitive achievements in addressing benchmark functions and power system challenges. Wang et al.^35^ proposed GOjDE, a parallelized differential evolution algorithm that leverages graphics processing units (GPUs) for solving complex high-dimensional global optimization problems. This amalgamation incorporates self-adaptive control parameters and GOBL to bolster solution quality and significantly reduce computational time. Zhao et al.^36^ elucidated SCE-OBL, an improved variant of the shuffled complex evolution algorithm, customized with OBL components. This adaptation is adeptly tailored to address the intricate permutation flow shop scheduling problem (PFSP), demonstrating enhanced convergence speed and solution quality while outperforming alternative algorithms on conventional instances. Luong et al.^37^ devised OMODE, an opposition-based multiple objective differential evolution algorithm, tailored to facilitate trade-off optimization among project duration, cost, and quality within construction management. This model demonstrates its proficiency in generating non-dominated solutions, offering an alternative avenue for achieving optimal time–cost-quality equilibrium. Pham et al.^38^ introduced the novel sine cosine algorithm (nSCA), which incorporates the OBL to enhance global optimization capabilities, and after rigorous testing against classical and CEC2017 benchmark functions, as well as practical applications, nSCA consistently outperforms other state-of-the-art optimization algorithms, proving its efficacy in addressing both theoretical and real-world optimization challenges.

### Research gap

Numerous advancements have been pursued to elevate the performance of GOA, encompassing diverse strategies such as binary variations^39,40^, chaotic adaptations^41,42^, the integration of the Levy flight mechanism^43,44^, and amalgamations with alternative algorithmic frameworks^45–47^. However, a coherent integration of both the TS and OBL methodologies, which seeks to strike an optimal balance between exploration and exploitation phases, remains an avenue less traveled. Such a comprehensive integration is poised to set the stage for global optimization. Given the current research landscape, our investigation rises to bridge this gap, endeavoring to harness the collective potential of the TS and OBL methodologies. Moreover, while the efficacy of MOGOA has been acknowledged in varied domains, its applications tailored to optimization dilemmas in the construction sector, particularly those centered on time, cost, and carbon dioxide emission trade-offs, are still in their infancy. By intertwining TS and OBL within the MOGOA paradigm to address these trade-offs, this study not only strives to plug an existing research gap but also aims to introduce a sophisticated tool tailored to these specific optimization challenges.

**Figure 1:**
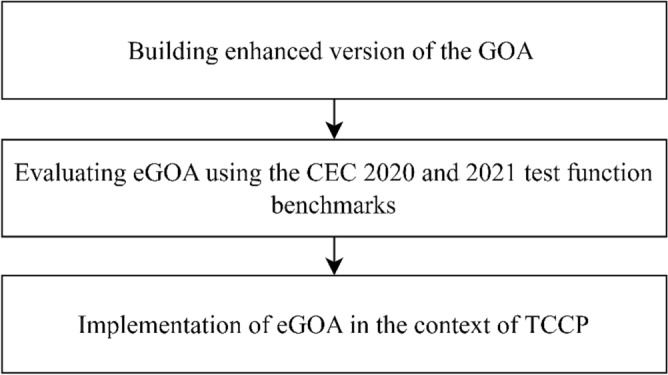
Process of implementation of the conceptual model.

This study presents an enhanced version of the MOGOA to bolster its optimization capacities in addressing the tri-objective challenge involving time, cost, and CO2 emissions. The research endeavours to bridge the gap in the existing literature that has been left by preceding investigations. Figure 1 illustrates the sequential stages involved in putting the conceptual model into practice.

## Optimization model development

This section is dedicated to introducing the proposed model, known as the eMOGOA, which amalgamates the MOGOA with the TS and OBL mechanisms. To facilitate a comprehensive understanding of this model, the indices, parameters, and decision-making variables are first defined. These elements are fundamental to the decision-making process within the eMOGOA framework and are detailed in “List of symbols” section.

### Grasshopper optimization algorithm (GOA)

In the wild, grasshoppers demonstrate the capacity to locate food sources and aggregate in groups for movement and reproduction. A distinctive characteristic of GOA is its computation of positions and velocities for "virtual grasshoppers" within the search space, all aimed at optimizing the objective function value of the given problem. The mathematical model to update the position of grasshoppers is presented as follows:

6 $${X}_{i}={S}_{i}+{G}_{i}+{A}_{i}$$

*X*_*i*_ is the position of the *i*th grasshopper; *S*_*i*_ is the social interaction, signifying the interplay between the ith grasshopper and others within the swarm; *G*_*i*_ is the gravitational attraction exerted on the *i*th grasshopper; *A*_*i*_ is the influence of wind and air circulation on the *i*th grasshopper.

It's worth noting that to incorporate stochastic behaviour, the equation can be formulated as follows:

7 $${X}_{i}={{r}_{1}S}_{i}+{r}_{2}{G}_{i}+{r}_{3}{A}_{i}$$

*r*_*1*_ is the random numbers within the range of [0, 1]; *r*_*2*_ is the random numbers within the range of [0, 1]; *r*_*3*_ is the random numbers within the range of [0, 1].

The S component in Eq. (6) is evaluated using the subsequent expression:

8 $${S}_{i}=\sum_{\begin{array}{c}j=1\\ j\ne 1\end{array}}^{N}s({d}_{ij})\widehat{{d}_{ij}}$$

*d*_*ij*_ is the distance between the *i*th and the *j*th grasshopper, calculated as follows: ${d}_{ij}=\left|{x}_{j-}{x}_{i}\right|$; *s* is the strength of social interaction forces; $\widehat{{d}_{ij}}=\frac{{x}_{j-}{x}_{i}}{{d}_{ij}}$ is the unit vector from the *i*th grasshopper to the jth grasshopper.

The magnitude of social interaction forces is determined by the function *s*, which is computed according to the following expression:

9 $$s\left(r\right)=f{e}^{\frac{-r}{l}}-{e}^{-r}$$

*f* is the strength of social interaction attraction, which impacts the extent of interplay and mutual attraction among grasshoppers; *l* is the rate at which the force of social interaction diminishes with distance; *r* is the distance value.

The G component in Eq. (6) is evaluated using the subsequent expression:

10 $${G}_{i}=-g\widehat{{e}_{g}}$$

*g* is the gravitational constant; $\widehat{{e}_{g}}$ is the unit vector directed towards the Earth's center.

The A component in Eq. (6) is evaluated using the subsequent expression:

11 $${A}_{i}=u\widehat{{e}_{w}}$$

*u* is the constant drift; $\widehat{{e}_{w}}$ is the unit vector aligned with the direction of the wind.

By substituting S, G, and A into the expression provided in Eq. (6), the formula can be expanded as follows:

12 $${X}_{i}=\sum_{\begin{array}{c}j=1\\ j\ne 1\end{array}}^{N}s\left(\left|{x}_{j-}{x}_{i}\right|\right)\frac{{x}_{j-}{x}_{i}}{{d}_{ij}}-g\widehat{{e}_{g}}+u\widehat{{e}_{w}}$$

*s* is the strength of social interaction forces; *d*_*ij*_ is the distance between the *i*th *and* *the* *j*th grasshopper; *g* is the gravitational constant; $\widehat{{e}_{g}}$ is the unit vector directed towards the Earth's center; *u* is the constant drift; $\widehat{{e}_{w}}$ is the unit vector aligned with the direction of the wind.

In the context of the optimization algorithm, the utilization of Eq. (12) is intentionally avoided due to its tendency to restrict the algorithm's ability to thoroughly explore and exploit the nearby regions within the solution space^9^. This specific nymph grasshopper model has been intricately crafted to address a grasshopper swarm functioning within an unbounded space. It's worth noting that this mathematical model was not directly applied to solve optimization problems, as the grasshoppers swiftly converge to their comfort zones and the swarm does not converge towards a singular point. In response, a modified version of Eq. (12) is employed to effectively address optimization challenges:

13 $${X}_{i}^{d}=c(\sum_{\begin{array}{c}j=1\\ j\ne 1\end{array}}^{N}c\frac{u{b}_{d}-l{b}_{d}}{2}s(\left|{x}_{j}^{d}-{x}_{i}^{d}\right|)\frac{{x}_{j}-{x}_{i}}{{d}_{ij}})+\widehat{{T}_{d}}$$

*ub*_*d*_ is the upper limit; *lb*_*d*_ is the lower limit; $\widehat{{T}_{d}}$ is the desired value; *c* is the coefficient value.

To compute the subsequent grasshopper position, information encompassing the target's position, the current grasshopper's position, and the positions of all other grasshoppers is utilized. As depicted in Eq. (13), the subsequent position of an individual grasshopper is determined by a combination of its current location, the global best solution, and the positional data of all other search agents. This implies that the GOA requires the active participation of all search agents in shaping the trajectory of each individual grasshopper. Notably, the initial part of Eq. (13) takes into account the relative positioning of the current grasshopper in relation to its counterparts within the region. Conversely, the subsequent segment limits the extent of movement around the target location. This duality highlights the algorithm's pursuit of both extensive exploration and focused exploitation within the entire swarm centered around the target.

To clarify, the parameter *c*_*1*_ governs the degree of restraint imposed on grasshopper movements near the target, achieving a balanced equilibrium between exploration and exploitation across the collective swarm. In contrast, *c*_*2*_ contributes to the contraction of attraction, comfort, and repulsion zones among grasshoppers, effectively reducing the spatial extent. As a result, *c*_*2*_ guides the grasshoppers in navigating the search space towards the optimal solution.

Noteworthy is the adaptive nature of *c*_*1*_, which progressively lessens the influence of repulsion and attraction forces among grasshoppers in proportion to the iteration count. Concurrently, *c*_*2*_ incrementally reduces the width of the comfort zone with increasing iterations. This strategic interplay emerges, where *c*_*1*_ refines exploitation during later optimization stages, and *c*_*2*_ gradually narrows the zones to enhance proximity to the optimal solution. Both *c*_*1*_ and *c*_*2*_ are unified as a single parameter, subject to adjustment as follows:

14 $$c={c}_{max}-l\frac{{c}_{max}-{c}_{min}}{L}$$

*c*_*max*_ is the upper limit of the parameter *c*; *c*_*min*_ is the lower limit of the parameter *c*; *l* is the current iteration; *L* is the total number of iterations.

### Tournament selection (TS)

**Figure 2:**
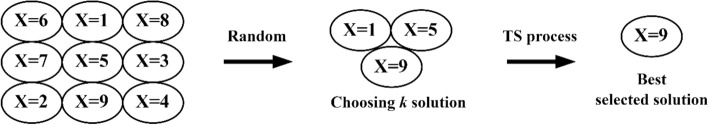
Tournament selection concept.

Figure 2 presents a visual representation that sheds light on the complexities of the TS process. In this methodology, a subset of *k* solutions is randomly sampled from the existing solution pool. Subsequently, the solution with the highest fitness score is identified and subsequently integrated into the population of the next generation. The magnitude of the tournament, referred to as the tournament size, plays a crucial role in this strategy. However, it is important to acknowledge that increasing the tournament size might result in a decrease in the expected diversity of the selected solutions^48^. In the context of this study, a tournament size of *k* = *5* is recommended as the optimal approach. The sets of solutions and their corresponding positions are systematically arranged within a matrix, as illustrated below:

15 $$X=\left[\begin{array}{c}{X}_{1}\\ \begin{array}{c}{X}_{2}\\ \dots \end{array}\\ {X}_{n}\end{array}\right]=\left[\begin{array}{ccc}\begin{array}{cc}{x}_{1}^{1}& {x}_{1}^{2}\end{array}& \dots & {x}_{1}^{d}\\ \begin{array}{cc}{x}_{2}^{1}& {x}_{2}^{2}\end{array}& \dots & {x}_{2}^{d}\\ \begin{array}{cc}\begin{array}{c}\dots \\ {x}_{n}^{1}\end{array}& \begin{array}{c}\dots \\ {x}_{n}^{2}\end{array}\end{array}& \begin{array}{c}\dots \\ \dots \end{array}& \begin{array}{c}\dots \\ {x}_{n}^{d}\end{array}\end{array}\right]$$

*X* is the set of solutions; *n* is the number of solutions; *d* is the number of dimensions.

In the context of the eMOGOA model, the TS mechanism finds its application in discerning the optimal choice from a collection of *k* solutions acquired from the solution pool. In the subsequent stages, Eq. (16) is invoked to facilitate the evolution of fitness across the entire range of solutions. By incorporating parameters extracted from the superior solution identified through TS, Eq. (16) establishes an environment where each solution is afforded the opportunity for advancement and enhancement of its fitness level. This pivotal phase assumes a paramount role in stimulating exploratory aspects and fostering a diverse assortment within the population.

16 $${x}_{i}^{j}=\left\{\begin{array}{c}{x}_{T}^{v} {r}_{4}<{C}_{T}\\ {x}_{i}^{v} {r}_{4}\ge {C}_{T}\end{array}\right.$$

${x}_{i}^{v}$ is the *v*th parameter of solution *i*; ${x}_{T}^{v}$ is the *v*th parameter of the solution selected through the *TS* process; *C*_*T*_ is the *TS* process condition.

subject to:

17 $${C}_{T}=0.1\times \left(1- \frac{l}{L}\right)$$

*r*_*4*_ is a randomly generated value within the range of 0 to 1.

### Opposition based learning (OBL)

**Figure 3:**
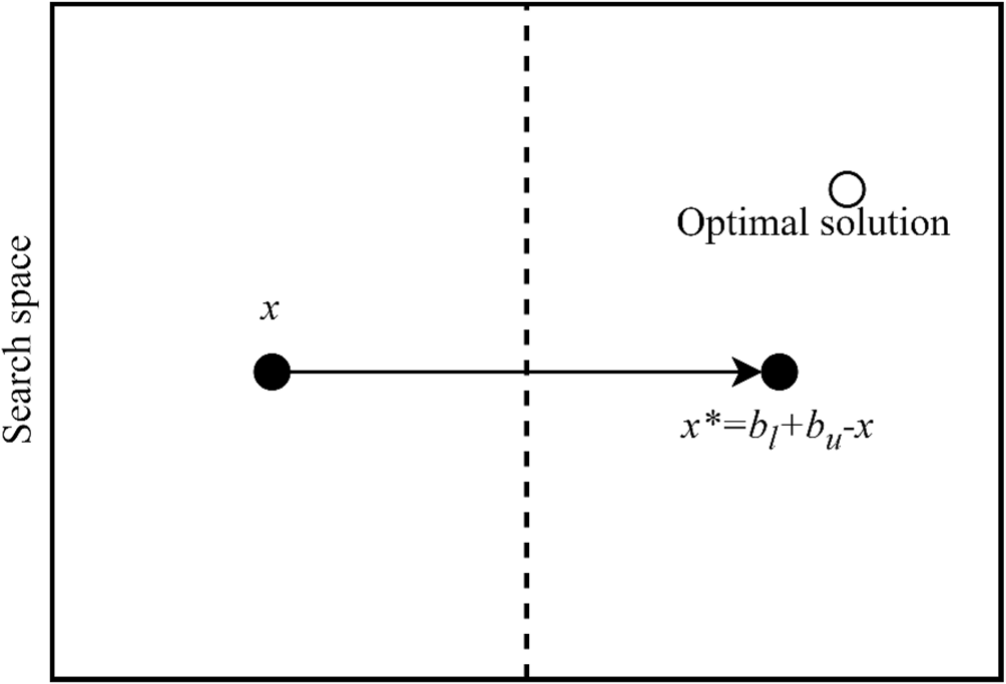
Opposition-based learning concept.

The principle of opposition-based learning is depicted in Fig. 3, where the opposing point X* is obtained by reflecting each coordinate of the initial point *X* across the midpoint situated between its lower and upper boundaries.

The concept of the opposition value x* for a number x within the interval *[b*_*l*_*,* *b*_*u*_*]* can be accurately described as follows:

18 $${x}^{*}={b}_{u}+{b}_{l}-x$$

*b*_*l*_ is the lower bound that define the range of *x.* *b*_*u*_ is the upper bound that define the range of *x.*

When extending this concept to higher dimensions, let's consider a point *X* with components *x*_*1*_, *x*_*2*_, …, *x*_*d*_ where each *x*_*v*_ falls within the interval [*b*_*l,v*_, *b*_*u,v*_]. Similarly, the opposition point X* with components ${x}_{1}^{*}, {x}_{2}^{*}, \dots , {x}_{d}^{*}$ can be defined for each dimension *j* as:

19 $${x}_{v}^{*}={b}_{u,v}+{b}_{l,v}-{x}_{v}$$

*b*_*l,v*_ is the lower bound associated with the *v*th dimension; *b*_*u,v*_ is the upper bound associated with the *v*th dimension.

During the optimization process, both the original point *X* and its corresponding opposite point X* are evaluated using the fitness function. Subsequently, a judicious decision is taken between these two solutions, favoring the superior one for retention and discarding the relatively inferior alternative.

### Proposed eMOGOA model for TCCP problems

**Figure 4:**
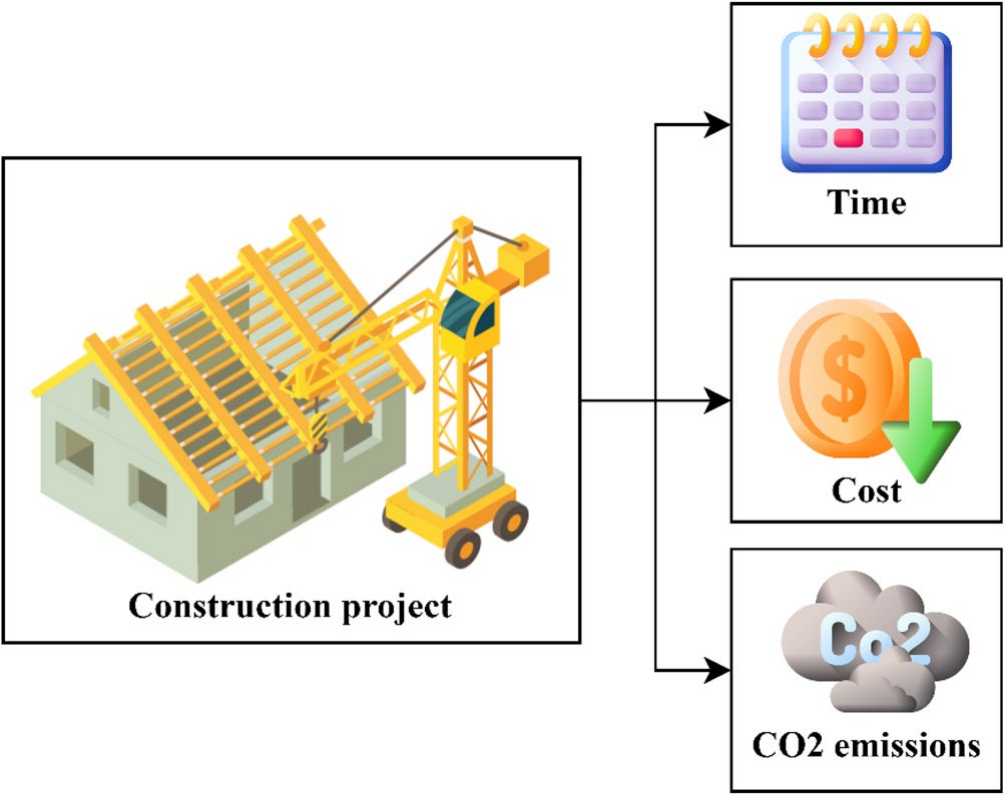
Time, cost, and carbon dioxide emission trade-off problems.

The optimization efforts within the TCCP framework are centrally focused on a tripartite minimization objective: the reduction of project duration, the management of costs, and the mitigation of carbon dioxide emissions, as depicted in Fig. 4. This multifaceted goal is achieved by meticulously selecting the most appropriate execution approach for each activity. Such a strategy ensures that every aspect of the project aligns with the overarching objectives of time efficiency, cost-effectiveness, and environmental sustainability.

**Figure 5:**
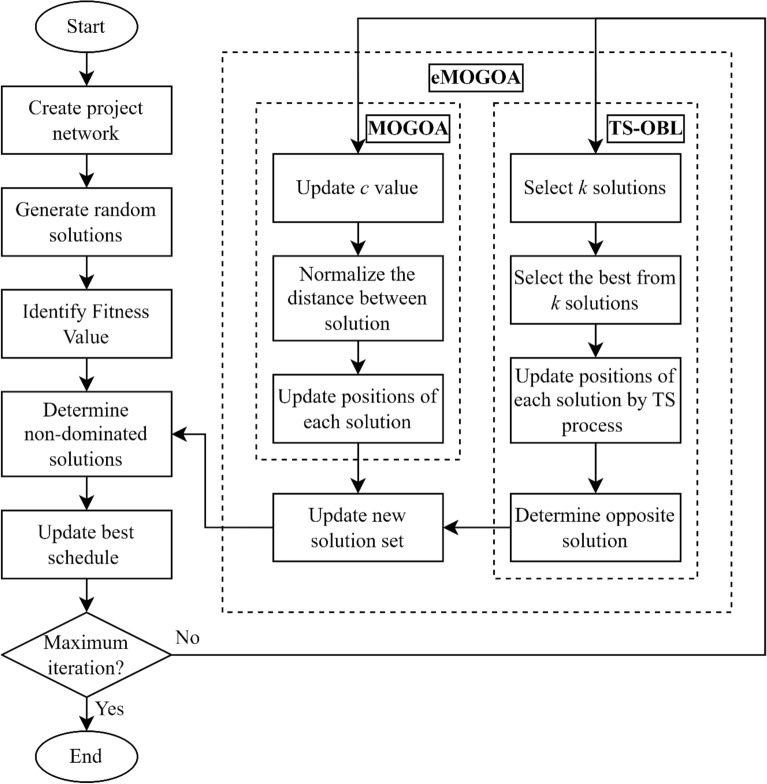
Flowchart of eMOGOA model for TCCP.

Table 2 provides the pseudo-code that outlines the eMOGOA model, which is a specialized solution designed for addressing the TCCP. This table offers a comprehensive explanation of the algorithm's operational framework. Furthermore, the algorithm's procedural sequence is visually depicted in Fig. 5, presented as a flowchart. This illustrative diagram provides a clear and step-by-step representation of the algorithm's progression.

**Table 2:** Pseudo-code of the eMOGOA model for TCCP.

**Input:** Number of solution (n) and maximum of iterations (L)
**Begin** Build project network; Generate randomized solution; **while** (stopping condition is not satisfied) **do** Determine fitness score (Eq. (15), Eq. (16) and Eq. (17)); ***Phase*** ***1:*** ***MOGOA*** ***process*** **[** Update c value using Eq. (8) **for** (each solution i) **do** Normalize the distances between solutions; Update the position of current solution by Eq. (8) **end** **]** ***Phase*** ***2:*** ***TS-OBL*** ***process*** **[** Determine the best solution through TS process **for** (each solution i) **do** Update the position of current solution by Eq. (11) Determine opposite solution of current solution by Eq. (14) Determine superior solution between current solution and opposite solution **end** **]** Determine non-dominated solutions Update new solution set **Return:** The best solution **End**
**Output:** The best solution and its score

The computational complexity of the eMOGOA is primarily influenced by three factors: the number of iterations (*L*), the number of solutions (*n*), and the number of dimensions (*d*). Within each iteration, the sorting of solutions is conducted, utilizing the Quicksort algorithm, which possesses a computational complexity of O(n^2^). Additionally, the TS mechanism, applied to every variable in every solution across iterations, has a complexity of *O*(log *n*). Consequently, the aggregate computational complexity of eMOGOA can be expressed as *O*(*L* × (*n*^2^ + *n* × *d* × log *n*)).

Despite the fact that this computational complexity may necessitate increased use of computational resources and result in more time consumption compared to the standard MOGOA, eMOGOA offers compensatory benefits. It ensures a balanced capability in exploring and exploiting the search space. Moreover, eMOGOA is adept at providing high-quality solutions for complex, real-world applications. These advantages underscore the utility of eMOGOA, particularly in scenarios where solution quality and robust search capabilities are paramount.

The performance of eMOGOA in TCCP was evaluated using several widely recognized evaluation metrics, as described below:

- C-metric (C)^49^ Given A and B as two distinct sets of solutions, the C-metric evaluates the degree of convergence between these sets, as described in Eq. (20). A result of *C*(*A*, *B*) equaling 1 indicates that each element within set A dominates every solution within set B. Conversely, a *C*(*A,* *B*) value of 0 suggests that none of the members of set B are dominated by those of set A. Owing to the asymmetric nature of the C-metric, calculating both *C*(*A,* *B*) and *C*(*B,* *A*) becomes imperative^50^.
  20 $$C\left(A,B\right)=\frac{\left|b\in B; \exists a\in A:a\le b\right|}{\left|B\right|}$$
  *A* and *B* is the two distinct sets of solutions; *a* is the solution in set *A*; *b* is the solution in set *B.*
- Diversification measurement (DM)^51^: This metric gauges the extent or dispersion of a non-dominated solution set, as defined in Eq. (21). In the context of this equation, *Min* *f*_*i*_ and *Max* *f*_*i*_ denote the minimum and maximum value of the *i*th objective function, respectively. In the realm of algorithms, a higher DM value holds greater merit.
  21 $$DM=\sqrt{\sum_{i}^{M}{\left(Min \,{f}_{i}-Max \,{f}_{i}\right)}^{2}}$$
  *Min* *f*_*i*_ is the minimum value of the *i*th objective function; *Max* *f*_*i*_ is the maximum value of the *i*th objective function.
- Mean Ideal Distance (MID)^52^: This metric measures the average proximity of solutions to a theoretical ideal point, as expressed in Eq. (22). Here, *N* stands for the number of chosen non-dominated solutions, 
  ${f}_{i}^{k}$ denotes the value of the objective function for the specific non-dominated set, and *M* is the count of distinct objective functions. An algorithm exhibiting a lower *MID* value is indicative of enhanced effectiveness.
  22 $$MID=\frac{\sum_{i=1}^{N}\sqrt{\sum_{k=1}^{M}{\left(\frac{{f}_{i}^{k}-{f}_{best}^{k}}{{f}_{total}^{k \,max}-{f}_{total}^{k \,min}}\right)}^{2}}}{N}$$
  *N* is the number of chosen non-dominated solutions; ${f}_{i}^{k}$ is the value of the objective function for the specific non-dominated set; *M* is the count of distinct objective functions.
- Spread (SP): This metric assesses the uniformity in the distribution of the obtained non-dominated solutions, as defined in Eq. (23). In this context, *d*_*i*_ represents the Euclidean distance between adjacent solutions, while *d* denotes the average distance among them. The terms *d*_*f*_ and *d*_*l*_ describe the Euclidean distances to the outermost solutions. An algorithm presenting a lower SP value is deemed desirable, and a score of zero reflects the optimal uniform distribution among the evaluated non-dominated solutions.
  23 $$SP=\frac{{d}_{f}+{d}_{l}+\sum_{i=1}^{N-1}|{d}_{i}-\overline{d }|}{{d}_{f}+{d}_{l}+(N-1)\overline{d} }$$
  *d*_*i*_ is the Euclidean distance between adjacent solutions; *d* is the average distance; *d*_*f*_ and *d*_*l*_ is the Euclidean distances to the outermost solutions.
- Hyper-volume (HV)^53^: this metric quantifies the spatial extent of the solutions, denoted as *X*, which is constrained at the top by a reference point, *W*. The line that joins the solution point *X*_*i*_ to the reference point *W* corresponds to the diagonal corners of each hypercube, represented as *v*_*i*_. The HV values are articulated in Eq. (24). An algorithm yielding a higher HV is considered superior. For standardization purposes, HV values are normalized between the range [0, 1], using the reference point.
  24 $$HV=volume(\bigcup_{i=1}^{|\Omega |}{v}_{i})$$
  *v*_*i*_ is the diagonal corners of each hypercube.

## Computational experiments

### CEC 2020 test functions

To assess the performance of the eMOGOA in solving multi-objective problems, a series of 15 test functions from the CEC 2020 suite, each possessing two or more objectives, were employed^54^. CEC 2020 is not only characterized by its multi-objective nature but also by its multi-modal properties, indicating that a single point on the Pareto front could be derived from multiple solutions within the decision space^55^. The specific details of the four benchmark problems utilized in our experiment are delineated in Table 3**.**

**Table 3:** CEC 2020 test functions.

Function	Name	Number of objectives	Number of variables
F1	MMF1	2	2
F2	MMF1_e	2	2
F3	MMF2	2	2
F4	MMF4	2	2
F5	MMF5	2	2
F6	MMF7	2	2
F7	MMF8	2	2
F8	MMF10	2	2
F9	MMF10_1	2	2
F10	MMF11	2	2
F11	MMF11_1	2	2
F12	MMF12	2	2
F13	MMF12_1	2	2
F14	MMF13	2	2
F15	MMF13_1	2	2

To guarantee a fair comparison among the algorithms, a standardized approach was adopted. For all the algorithms being evaluated, the number of iterations was uniformly set at 1000. Furthermore, the population size was fixed at 50, indicating the number of solutions generated in a random manner. Comprehensive assessment of each algorithm's performance was facilitated by conducting 30 independent runs, ensuring robustness and reliability in the evaluation process. A comparative analysis was conducted between eMOGOA and five well-known multi-objective algorithms: dragonfly algorithm (MODA), ant lion optimizer (MOALO), slime mold algorithm (MOSMA), particle swarm optimization (MOPSO) and the original MOGOA. The parameter settings of the different algorithms are shown in Table 4.

**Table 4:** Parameter settings of the different algorithms.

Algorithm	Parameter settings
eMOGOA	*c*_*max*_ = 1; *c*_*min*_ = 0.00001; *k* = 5
MOGOA	*c*_*max*_ = 1; *c*_*min*_ = 0.00001
MOPSO	*c*_*1*_ = 2; *c*_*2*_ = 2; *w*_*min*_ = 0.3; *w*_*max*_ = 0.7
MODA	*β* = 1.5
MOALO	*l*: current iteration; *L*: maximum number of iterations *w* = 2 when t > 0.1*L*; *w* = 3 when *l* > 0.5*L*; w = 4 when *l* > 0.75*L*; w = 5 when *l* > 0.9*L*; and *w* = 6 when *l* > 0.95*L*
MOSMA	*z* = 0.03

The Inverted generational distance (IGD)^55^ metric is utilized to evaluate the convergence properties of the algorithm. This measure involves a comparison between the Pareto optimal solutions generated by each algorithm and the true Pareto front. The calculation of IGD is based on the following equation:

25 $$IDG=\frac{\sum_{i=1}^{p}{d}_{i}}{p}$$

*n* is the total number of members or solutions present in the true Pareto front; *d*_*i*_ is the Euclidean distance between each member of the true Pareto front solutions and the nearest solution among the Pareto optimal non-dominated solutions obtained by the algorithm.

The results obtained by the eMOGOA, as well as MOGOA, MOPSO, MOALO, MODA, and MOSMA, on test functions ranging from F1 to F15, are systematically presented in Tables 5, 6 and 7. The performance efficacy of the multi-objective versions of eMOGOA was quantitatively assessed using two principal statistical indicators: *avg* and *std*. These indicators were calculated based on the IGD values derived from each algorithm across 30 independent runs. A comprehensive review of the data in Tables 5, 6 and 7 indicates a notably superior performance of the eMOGOA in many of the CEC 2020 test scenarios. Specifically, eMOGOA demonstrated an average IGD value that surpassed the multi-objective versions of MOGOA, MODA, and MOSMA in 13 out of the 15 functions and exceeded MOALO and MOPSO in 12 out of these 15 functions.

**Table 5:** IGD values obtained from various algorithms applied to functions F1 to F5.

Algorithm/function	Statistical metrics	*F1*	*F2*	*F3*	*F4*	*F5*
MOALO	*Avg*	0.016815	0.013208	0.003089	0.008327	0.016591
	*Std*	0.000077	0.000089	0.000238	0.000143	0.000108
MOPSO	*Avg*	0.010317	0.004323	0.008936	0.018429	0.009545
	*Std*	0.000629	0.001454	0.000844	0.000319	0.001876
MODA	*Avg*	0.011620	0.016257	0.012253	0.016875	0.012967
	*Std*	0.000068	0.000239	0.000265	0.000175	**0.000023**
MOSMA	*Avg*	0.011134	**0.002831**	0.012134	0.009014	0.013840
	*Std*	0.000029	0.000298	0.000182	0.000187	0.000205
MOGOA	*Avg*	0.008347	0.007868	0.016727	**0.007792**	0.013567
	*Std*	0.000273	**0.000020**	0.000274	0.000143	0.000263
eMOGOA	*Avg*	**0.006535**	0.007670	**0.002849**	0.011008	**0.008345**
	*Std*	**0.000022**	0.000155	**0.000155**	**0.000016**	0.000148

Significant values are in bold.

**Table 6:** IGD values obtained from various algorithms applied to functions F6 to F10.

Algorithm/function	Statistical metrics	*F6*	*F7*	*F8*	*F9*	*F10*
MOALO	*Avg*	0.018406	0.016436	0.004112	0.015735	**0.006654**
	*Std*	**0.000061**	0.000284	0.000149	0.000151	0.000228
MOPSO	*Avg*	0.006895	0.027835	0.008041	0.019925	0.019153
	*Std*	0.000393	0.001765	0.001142	0.001019	0.001982
MODA	*Avg*	**0.003284**	0.016682	0.011289	0.018771	0.015911
	*Std*	0.000243	0.000097	0.000212	0.000200	0.000093
MOSMA	*Avg*	0.019482	0.015743	0.005229	0.012130	0.013370
	*Std*	0.000175	**0.000034**	0.000177	0.000262	0.000168
MOGOA	*Avg*	0.013072	0.009402	0.008655	**0.003558**	0.016498
	*Std*	0.000206	0.000067	**0.000092**	0.000190	0.000068
eMOGOA	*Avg*	0.007863	**0.008127**	**0.003967**	0.008351	0.019244
	*Std*	0.000061	0.000166	0.000223	**0.000042**	**0.000025**

Significant values are in bold.

**Table 7:** IGD values obtained from various algorithms applied to functions F11 to F15.

Algorithm/function	Statistical metrics	*F11*	*F12*	*F13*	*F14*	*F15*
MOALO	*Avg*	0.014575	0.019917	0.011541	0.017030	0.004924
	*Std*	0.000087	0.000071	0.000102	0.000186	**0.000066**
MOPSO	*Avg*	0.015261	0.012541	0.013659	0.010075	0.011203
	*Std*	0.000342	0.000235	0.004939	0.002335	0.004890
MODA	*Avg*	0.011472	**0.002629**	0.012304	0.005470	0.013644
	*Std*	0.000060	0.000036	**0.000062**	0.000082	0.000085
MOSMA	*Avg*	0.003498	0.004464	0.019130	0.004891	0.010293
	*Std*	0.000160	0.000220	0.000106	**0.000062**	0.000232
MOGOA	*Avg*	0.006912	0.011465	0.007088	0.013593	0.014615
	*Std*	0.000203	0.000089	0.000278	0.000135	0.000285
eMOGOA	*Avg*	**0.002535**	0.016085	**0.003857**	**0.006551**	**0.004733**
	*Std*	**0.000018**	**0.000032**	0.000234	0.000065	0.000111

Significant values are in bold.

Data presented in Tables 5, 6 and 7 clearly demonstrate that the enhanced version of the eMOGOA significantly outperforms the original MOGOA in identifying higher-quality solutions. These tables also highlight the improved capability of eMOGOA in locating optimal global solutions and its adeptness at avoiding local peaks. The superior performance of eMOGOA can be attributed to the integration of TS and OBL strategies. The incorporation of TS and OBL into eMOGOA plays a crucial role in introducing variability into solution vectors, essential for navigating complex search landscapes. Furthermore, these strategies assist in retaining superior solutions by facilitating a comparative analysis of fitness values between original and OBL-derived solutions. This feature enables eMOGOA to discern areas within the search field that are rich in potential, thereby ensuring comprehensive exploration and the eventual identification of optimal solutions.

### Time, cost, and carbon dioxide emission trade-off problems (TCCP)

To validate the efficacy and proficiency of the proposed model in addressing the TCCP challenge, an in-depth analysis of a real-world construction case study was undertaken. The selected case study focuses on a highway construction project that encompasses 29 unique activities. It's worth noting that the activity data provided is both quantitative and highly accurate. For clearer understanding, Table 8 visually displays the pertinent data from the case study. This table gives a detailed overview of the interdependencies between activities, their durations, costs, and carbon dioxide emissions—all of which are vital aspects of the available execution alternatives.

**Table 8:** Data of the 29-activity project.

No	Logical	Option 1			Option 2			Option 3		
		T	C	CE	T	C	CE	T	C	CE
1	–	5	2030	175.43	4	2300	172.73	–	–	–
2	1FS − 3 days	8	1020	106.28	7	1280	93.2	6	1510	86.11
3	1, 2	8	1700	236.25	7	1850	229.24	6	2090	200.88
4	3	4	590	197.92	3	730	193.97	–	–	–
5	4SS + 1 day	2	90	140.53	–	–	–	–	–	–
6	1	4	910	117.33	3	1100	115.89	–	–	–
7	2, 6FS − 1 day	2	250	112.08	–	–	–	–	–	–
8	7, 3	7	1490	175.52	6	1650	160.49	5	1830	144.46
9	4, 8	4	520	132.87	3	750	119.33	–	–	–
10	5, 9FF + 1 day	2	90	87.32	–	–	–	–	–	–
11	5, 10	1	50	92.24	–	–	–	–	–	–
12	11	8	3260	129.99	7	3580	129.61	6	3710	105.67
13	12SS + 2 days	5	1140	244.36	4	1400	239.02	3	1720	199.47
14	13SS + 2 days	4	300	85.87	3	450	76.27	–	–	–
15	12FS − 4 days,14FS − 2 days	8	1020	131.34	6	1300	129.81	5	1430	99.65
16	5FS − 6 days	9	790	162.27	8	900	148.99	6	1180	147.82
17	15SS + 4 days	13	3340	166.84	12	3750	159.92	11	4060	120.91
18	15FS + 4 days	9	470	196.37	8	650	179.28	7	830	172.83
19	15	6	460	201.71	5	600	190.75	4	810	153.89
20	17SS + 3 days	6	1280	112.05	5	1430	94.8	–	–	–
21	20SS + 2 days	14	1090	218.89	12	1320	204.64	10	1560	167.94
22	21SS + 2 days	14	900	180.82	11	1140	178.3	9	1400	172.48
23	22FS − 9 days	14	2220	200.77	12	2510	192.33	11	2690	183.89
24	23SS + 6 days	3	230	196.33	–	–	–	–	–	–
25	23FS − 4 days	6	1590	106.57	5	1790	95.07	4	1990	59.81
26	25SS + 4 days	10	2630	93.86	9	2930	82.05	8	3240	49.94
27	26	8	2060	174.75	7	2450	162.6	6	2660	125.81
28	27FS − 3 days	10	320	164.01	9	440	153.11	8	610	129.57
29	28	1	50	193.07	–	–	–	–	–	–

**Figure 6:**
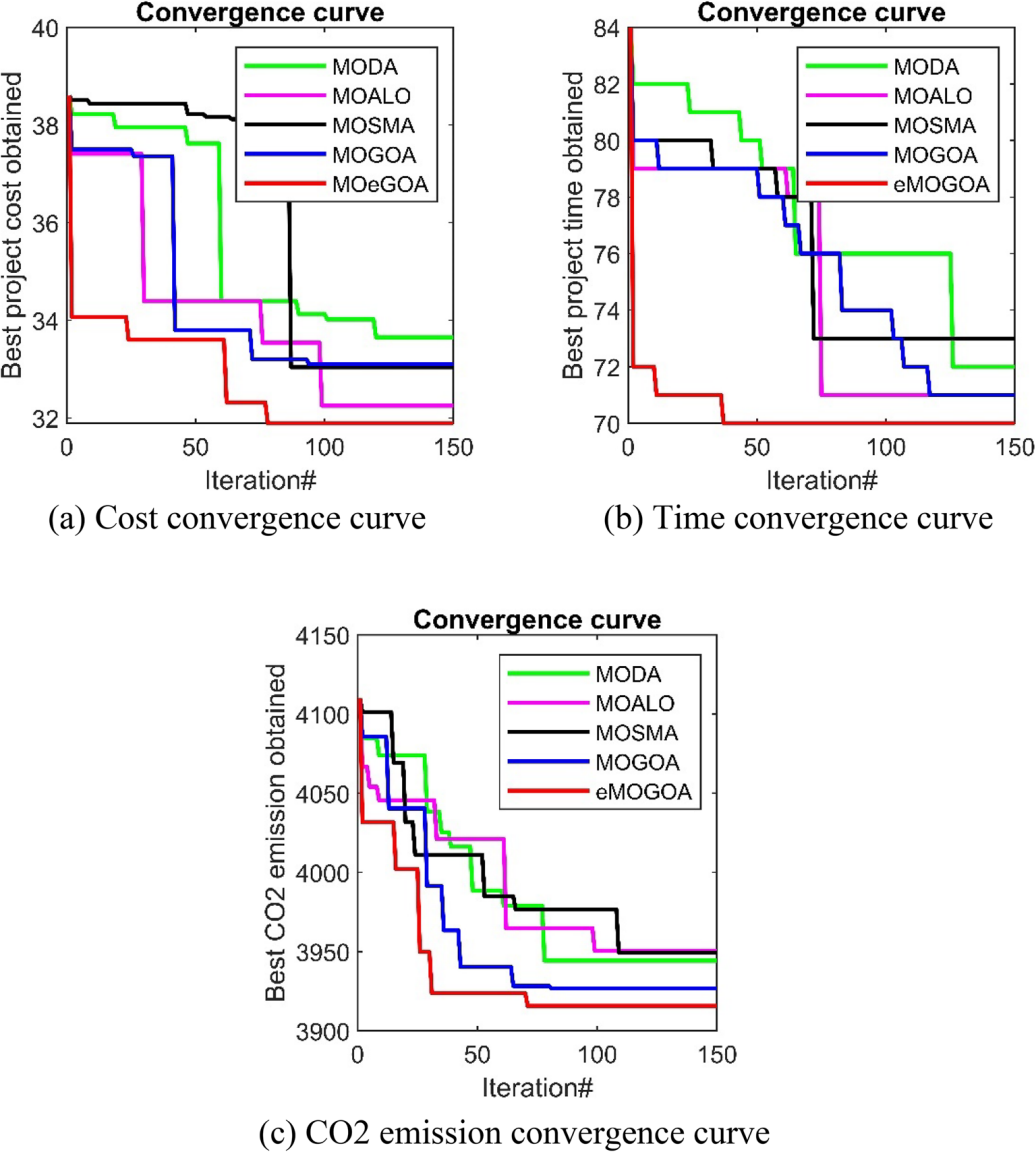
Convergence curve of different algorithm.

A comparative analysis was conducted between eMOGOA and four well-known mutil-objective algorithms: MODA, MOALO, MOSMA, and the original MOGOA. Specific performance metrics were used, with a population size standardized at 100 and a maximum iteration limit set at 150. Thirty trials were conducted for each algorithm. The most optimal outcomes from these trials were then analyzed to assess the optimization efficiency across the algorithms, ensuring an objective and balanced evaluation was maintained. In Fig. 6, the convergence curves for three objectives: cost, duration, and CO2 emission, are displayed, illustrating the performance of MODA, MOALO, MOSMA, MOGOA, eMOGOA. The top non-dominated solutions, as determined by the different algorithms, are presented in Tables 9, 10, and 11. From the empirical evidence, a clear advantage for eMOGOA over MODA, MOALO, MOSMA, and MOGOA in all evaluated objectives was observed. Furthermore, eMOGOA can determine solutions with shorter project durations compared to MOSGO^25^. In the context of cost and carbon emission, eMOGOA and MOSGO produce competitive results. However, eMOGOA demonstrates computational efficiency, requiring evaluations of only 12,500 evaluation functions within the solution space to identify the optimal solution. In contrast, MOSGO demands evaluations for up to 15,000 options^25^.

**Table 9:** Best non-dominated solutions obtained by different algorithms (sorted by time).

Algorithm	Solution	Time (days)	Cost (1000 USD)	CO_2_ emission (kg)
MOSGO^25^	[2 3 3 1 1 1 1 1 1 1 1 1 1 2 1 1 1 1 1 1 1 3 3 1 1 3 3 3 1]	**73**	35.66	4313.28
	[2 3 3 1 1 1 1 1 1 1 1 1 1 2 1 1 1 1 1 1 1 3 3 1 1 2 3 3 1]	**74**	35.35	4345.39
MODA	[1 3 3 1 1 1 1 3 1 1 1 2 1 2 1 1 3 1 1 2 1 3 3 1 1 3 3 3 1]	**72**	36.92	4221.36
	[2 1 3 1 1 1 1 3 2 1 1 1 1 2 2 1 2 1 2 1 2 3 3 1 3 3 3 3 1]	**72**	37.2	4208.43
MOALO	[2 3 3 1 1 2 1 3 2 1 1 3 3 1 1 1 3 1 3 1 1 3 3 1 2 3 3 3 1]	**71**	38.57	4102.38
	[2 3 3 1 1 2 1 3 2 1 1 3 2 1 2 2 2 1 2 2 3 3 3 1 3 3 3 3 1]	**71**	38.94	4099.53
MOSMA	[1 3 3 2 1 2 1 1 2 1 1 2 3 2 2 1 3 3 3 2 1 3 3 1 1 3 3 3 1]	**73**	38.71	4115.71
	[1 3 1 2 1 2 1 3 2 1 1 2 3 2 3 3 2 2 2 1 3 3 3 1 3 3 3 3 1]	**73**	39.2	4077.27
MOGOA	[1 3 3 2 1 1 1 3 2 1 1 1 3 2 2 2 1 2 1 2 3 3 3 1 2 3 3 3 1]	**71**	38.07	4110.94
	[2 3 3 1 1 1 1 3 1 1 1 1 3 2 2 3 1 1 3 1 1 3 3 1 2 3 3 3 1]	**71**	37.8	4162.03
eMOGOA	**[2** **3** **3** **2** **1** **2** **1** **3** **2** **1** **1** **1** **1** **2** **1** **2** **1** **1** **1** **1** **1** **3** **3** **1** **1** **3** **3** **3** **1]**	**70**	**36.67**	**4250.01**
	**[2** **3** **3** **2** **1** **1** **1** **3** **2** **1** **1** **3** **3** **2** **3** **3** **3** **3** **3** **2** **2** **3** **3** **1** **3** **3** **3** **3** **1]**	**70**	**40.37**	**3918.3**

Significant values are in bold.

**Table 10:** Best non-dominated solutions obtained by different algorithms (sorted by cost).

Algorithm	Solution	Time (days)	Cost (1000 USD)	CO_2_ emission (kg)
MOSGO^25^	[1 1 1 1 1 1 1 1 1 1 1 1 1 1 1 1 1 1 1 1 1 1 1 1 1 1 1 1 1]	93	**31.89**	4533.7
	[1 1 1 1 1 1 1 1 1 1 1 1 1 1 1 1 1 1 1 1 1 1 1 1 1 1 1 2 1]	92	**32.02**	4522.8
MODA	[1 1 1 1 1 2 1 1 1 1 1 3 1 1 1 1 3 1 1 1 1 1 1 1 3 1 1 1 1]	93	**33.65**	4415.19
	[1 1 1 1 1 2 1 1 1 1 1 3 2 1 1 2 1 1 1 2 3 1 1 1 2 1 1 1 1]	93	**33.72**	4409.56
MOALO	[1 1 1 1 1 1 1 1 1 1 1 1 1 1 1 1 1 3 1 1 1 1 1 1 1 1 1 1 1]	93	**32.25**	4510.1
	[1 1 1 1 1 1 1 1 1 1 1 1 1 1 1 1 1 1 1 2 2 1 1 1 1 1 1 1 1]	93	**32.27**	4502.14
MOSMA	[1 1 1 2 1 1 1 1 1 1 1 1 1 1 1 3 1 1 1 2 3 1 1 1 1 1 1 1 1]	93	**33.04**	4447.04
	[1 1 1 1 1 1 1 1 1 1 1 1 2 1 3 2 1 1 2 2 2 1 1 1 1 1 1 1 1]	93	**33.19**	4440.87
MOGOA	[1 1 1 2 1 1 1 1 1 1 1 1 1 1 3 2 1 1 3 1 1 1 1 1 2 1 1 1 1]	93	**33.1**	4425.4
	[1 1 1 1 1 1 1 1 1 1 1 1 1 1 1 1 1 1 3 1 3 1 1 1 3 1 1 1 1]	93	**33.11**	4388.11
eMOGOA	**[1** **1** **1** **1** **1** **1** **1** **1** **1** **1** **1** **1** **1** **1** **1** **1** **1** **1** **1** **1** **1** **1** **1** **1** **1** **1** **1** **1** **1]**	**93**	**31.89**	**4533.64**
	**[1** **1** **1** **1** **1** **1** **1** **1** **1** **1** **1** **1** **1** **1** **1** **1** **1** **1** **1** **1** **1** **1** **1** **1** **1** **1** **1** **2** **1]**	**92**	**32.02**	**4522.74**

Significant values are in bold.

**Table 11:** Best non-dominated solutions obtained by different algorithms (sorted by CO_2_ emission).

Algorithm	Solution	Time (days)	Cost (1000 USD)	CO_2_ emission (kg)
MOSGO^25^	[2 3 3 2 1 2 1 3 2 1 1 3 3 2 3 3 3 3 3 2 3 3 3 1 3 3 3 3 1]	70	40.84	**3915.7**
	[2 3 3 2 1 2 1 3 2 1 1 3 3 2 3 2 3 3 3 2 3 3 3 1 3 3 3 3 1]	70	40.37	**3916.9**
MODA	[2 1 3 2 1 2 1 3 2 1 1 3 3 2 3 3 3 3 3 2 3 3 2 1 3 3 3 3 1]	73	40.17	**3944.3**
	[2 3 3 1 1 2 1 3 2 1 1 3 3 2 3 1 3 3 3 2 3 3 1 1 3 3 3 3 1]	73	39.84	**3950.97**
MOALO	[1 3 3 2 1 1 1 3 1 1 1 3 3 2 3 3 3 3 3 1 3 3 3 1 3 3 3 3 1]	72	40	**3950.62**
	[2 1 3 1 1 1 1 3 2 1 1 3 3 2 3 1 3 3 3 2 3 3 3 1 3 3 3 3 1]	72	39.63	**3955.7**
MOSMA	[2 1 3 2 1 2 1 3 1 1 1 3 3 2 3 3 3 3 3 2 3 3 3 1 3 3 3 3 1]	73	40.12	**3949.4**
	[2 3 3 1 1 1 1 3 2 1 1 3 3 2 3 1 3 3 3 2 3 3 1 1 3 3 3 3 1]	73	39.65	**3952.41**
MOGOA	[2 3 3 2 1 1 1 3 2 1 1 3 3 1 3 3 3 3 3 2 3 3 3 1 3 3 3 3 1]	71	40.5	**3926.73**
	[2 3 3 1 1 2 1 3 2 1 1 3 3 1 3 2 3 3 3 2 3 3 3 1 3 3 3 3 1]	71	40.27	**3930.41**
eMOGOA	**[2** **3** **3** **2** **1** **2** **1** **3** **2** **1** **1** **3** **3** **2** **3** **3** **3** **3** **3** **2** **3** **3** **3** **1** **3** **3** **3** **3** **1]**	**70**	**40.84**	**3915.69**
	**[2** **3** **3** **2** **1** **1** **1** **3** **2** **1** **1** **3** **3** **2** **3** **2** **3** **3** **3** **2** **3** **3** **3** **1** **3** **3** **3** **3** **1]**	**70**	**40.37**	**3918.3**

Significant values are in bold.

From Table 9, which categorizes the optimal non-dominated solutions by duration, eMOGOA stands out with the shortest recorded duration of 70 days. Similarly, Table 10, which focuses on cost considerations, highlights eMOGOA's cost-effectiveness, clocking the lowest expenditure at 32,030 USD. On the environmental front, Table 11, based on CO2 emissions, showcases eMOGOA's eco-friendliness, registering emissions of just 3918.3 kg.

**Figure 7:**
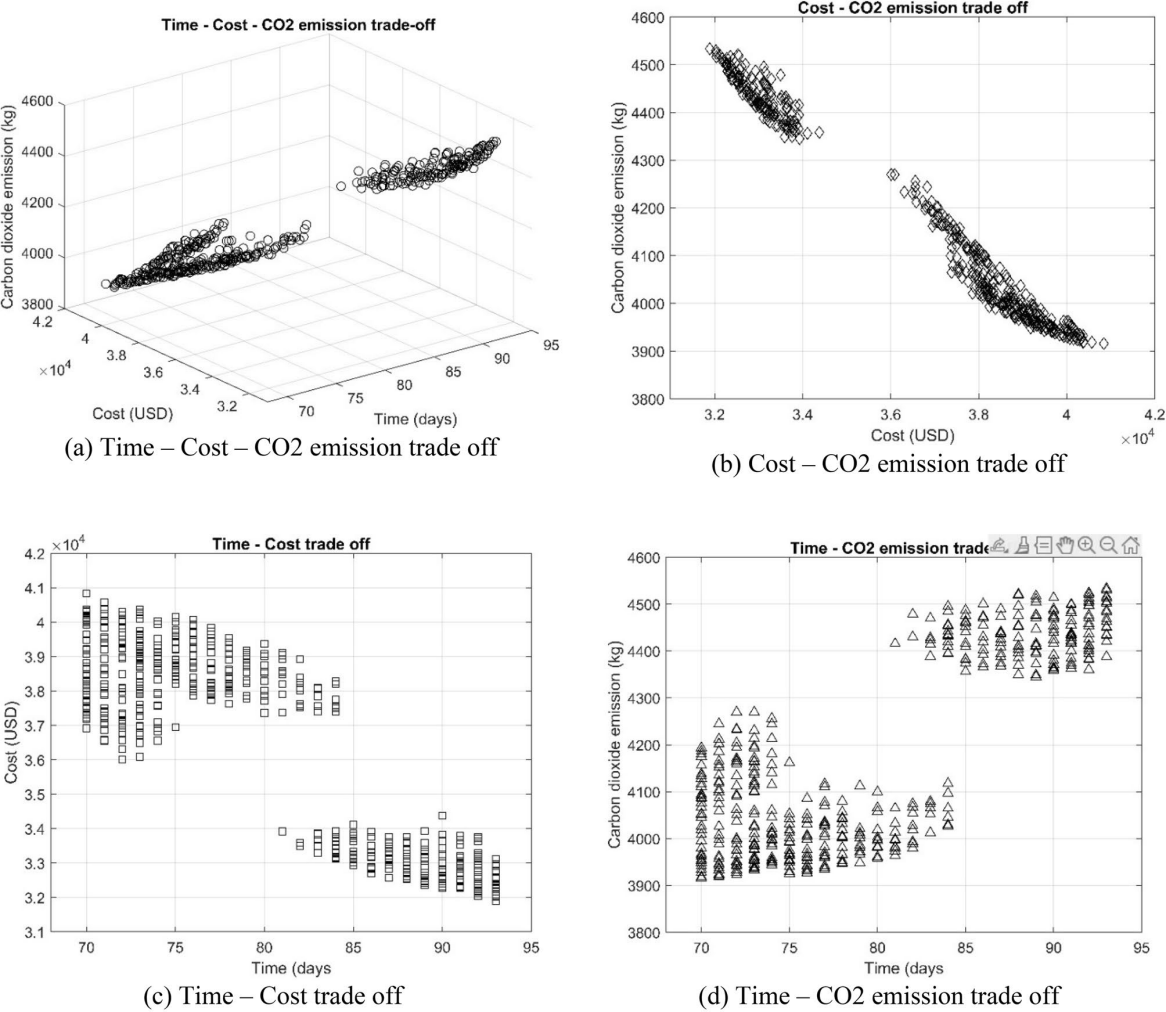
Pareto front obtained by eMOGOA model for TCCP.

The Pareto front is a concept from multi-objective optimization, and it represents the set of solutions that are not dominated by any other solutions when considering multiple objectives. In other words, the Pareto front consists of solutions that are optimal in the sense that no other solution is better in all objectives. An important aspect of the Pareto front is that there is no single "best" solution, as the optimal solution depends on the trade-offs one is willing to make between the objectives. By examining the Pareto front, decision-makers can choose the solution that best aligns with their preferences or priorities. Figure 7 illustrates the relationship among time, cost, and CO2 emission objectives. Specifically, Fig. 7a depicts the Pareto frontier determined by the three objectives, as represented by the eMOGOA. To further highlight the intricate interplay between any two of these objectives, non-dominated solutions are projected onto a two-dimensional plane, as shown in Fig. 7b–d. For example, Fig. 7c explores the complex relationship between project duration and associated costs.

Table 12 delivers an insightful comparison of the eMOGOA (labelled as A1) with established benchmark algorithms, notably MOGOA (A2), MODA (A3), MOALO (A4), and MOSMA (A5). Across the discussed scenarios, eMOGOA consistently stands out, significantly outpacing its counterparts. The table systematically presents results for these five algorithms in terms of the C-metric, elucidating their best, worst, average, and standard deviation values. Within the assessed problems, eMOGOA, on average, surpasses the solutions formulated by MOGOA, MODA, MOALO, and MOSMA, boasting dominance percentages of 87%, 76%, 73%, and 69%, correspondingly.

**Table 12:** C-metrics obtained by different algorithms.

Performance measurement	C(A1, A2)	C(A2, A1)	C(A1, A3)	C(A3, A1)	C(A1, A4)	C(A4, A1)	C(A1, A5)	C(A5, A1)
Best	0.96	0.16	0.96	0.32	0.84	0.20	0.88	0.32
Worst	0.72	0.00	0.52	0.04	0.56	0.04	0.56	0.16
Average	0.87	0.07	0.76	0.18	0.73	0.15	0.69	0.22
Standard deviation	0.06	0.03	0.11	0.08	0.07	0.04	0.08	0.04

Table 13 showcases the comparative average results of multiple algorithms using DM, MID, SP, and HV metrics. Upon scrutinizing Table 13, it is evident that eMOGOA outperforms competing algorithms, notably MOGOA, MODA, MOALO, MOSMA, MOSGO^25^, MODE^25^ and MOPSO^25^. Analyzing the average results, eMOGOA clearly stands out, marking the highest DM value at 29.106. Furthermore, eMOGOA claims the zenith HV value, set at 0.899. The proposed algorithm also distinguishes itself by pinpointing solutions with the lowest MID and SP values, marked distinctly at 0.764 and 0.416, respectively.

**Table 13:** The average experimental outputs of different algorithms.

Algorithm	DM	MID	SP	HV
eMOGOA	**29.106**	**0.764**	**0.416**	**0.899**
MOGOA	19.891	1.126	0.579	0.801
MODA	15.002	1.478	0.786	0.781
MOALO	18.129	1.383	0.700	0.731
MOSMA	20.964	0.901	0.666	0.827
MOSGO^25^	26.113	0.872	0.462	0.875
MODE^25^	20.176	1.012	0.523	0.803
MOPSO^25^	14.371	1.123	0.721	0.706

Significant values are in bold.

## Discussion

The grasshopper optimization algorithm (MOGOA) is inspired by the swarming behaviour of grasshoppers in nature. This optimization method is adeptly designed for diverse applications, encompassing fields from engineering and science to economics. By harnessing the core principles of grasshopper swarming behaviour, MOGOA effectively explores and identifies optimal solutions. However, for complex optimization challenges characterized by multidimensionality and multimodality, MOGOA can encounter convergence issues, sometimes gravitating toward local optima.

To address these limitations, this study introduces the enhanced grasshopper optimization algorithm (eMOGOA). This advanced technique integrates opposition-based learning (OBL) with the tournament selection (TS) approach, thereby amplifying the explorative capabilities of the original MOGOA. The synergistic interaction between OBL and TS not only allows for rapid transitions in solution vectors but also ensures the retention of high-quality solutions. This is achieved through a comparative analysis of fitness values between original and OBL-enhanced solutions. The effectiveness of eMOGOA has been rigorously tested alongside established algorithms such as MODA, MOALO, MOSMA, MOPSO, and the foundational MOGOA, using the CEC 2020 test function benchmarks.

- In the realm of construction project management, success is often defined by key benchmarks including time, cost, and quality. However, environmental factors, notably carbon dioxide (CO2) emissions linked to construction material production, are frequently overlooked. Recognizing this gap, this research presents the eMOGOA as a novel method to address the trade-offs between time, cost, and CO2 emissions (TCCP) in construction projects.
- The results indicate that eMOGOA outperforms other algorithms, showcasing dominant performance metrics compared to MOGOA, MODA, MOALO, and MOSMA. Specifically, eMOGOA boasts dominance percentages of 87%, 76%, 73%, and 69% on C-metric values.
- Furthermore, when benchmarked against algorithms such as MOGOA, MODA, MOALO, MOSMA, MOSGO^25^, MODE^25^ and MOPSO^25^, eMOGOA consistently excelled, achieving the highest DM value at 29.106 and HV value at 0.899. Concurrently, it recorded the lowest MID and SP values at 0.764 and 0.416, respectively.
- The proposed model has demonstrated its efficacy in reducing time, cost, and CO2 emissions, positioning itself as a promising solution for optimization challenges within the construction industry.

## Conclusion

By integrating the opposition-based learning (OBL) method with the tournament selection (TS) approach, this study introduces eMOGOA, an enhanced technique poised to amplify the explorative capabilities of MOGOA. The orchestrated interplay between the OBL and TS mechanisms facilitates not only abrupt transitions in solution vectors but also the retention of high-quality solutions through the comparison of fitness values between original and OBL-enhanced solutions. This unique characteristic positions eMOGOA to adeptly identify areas of high potential within the search landscape. The efficacy of eMOGOA is subjected to rigorous scrutiny in parallel with established algorithms such as MODA, MOALO, MOSMA, MOPSO, and the foundational MOGOA, using the CEC 2020 test function benchmarks. Moreover, the versatility and prowess of eMOGOA manifest in its aptitude to adeptly navigate the intricacies of time, cost, and carbon dioxide emission trade-off problems (TCCP).

- The eMOGOA demonstrated dominant performance metrics in comparison to standard MOGOA, MODA, MOALO, and MOSMA. Notably, eMOGOA exhibited dominance percentages of 87%, 76%, 73%, and 69% on C-metric values, respectively, indicating its superior efficiency in optimization tasks.
- In benchmarks against a broader range of algorithms, including MOGOA, MODA, MOALO, MOSMA, MOSGO^25^, MODE^25^ and MOPSO^25^, the eMOGOA consistently outperformed. It achieved the highest DM value at 29.106 and HV value at 0.899. Additionally, eMOGOA recorded the lowest MID and SP values at 0.764 and 0.416, respectively.

Analytical evaluations underscore the superiority of eMOGOA over several metaheuristic strategies, highlighting its amplified capacity to unearth optimal solutions in both benchmark and real-world optimization contexts. Such revelations bolster the prominence of eMOGOA in the realm of engineering optimization, setting a new precedent for sophisticated problem-solving and strategic decision-making processes. Grounded in empirical evidence, eMOGOA stands out as a formidable and dependable instrument, primed to tackle diverse optimization impediments prevalent in practical scenarios. However, eMOGOA has some limitations, including the need for proper parameter tuning, the tendency to get stuck in local optima, and slow convergence. The algorithm's selection process relies on the Pareto dominance relation among different solutions, and as the number of non-dominant points increases, the archive fills up quickly, leading to slower optimization. Increasing the size of the archive to accommodate more objectives may hinder the algorithm's ability to converge to the true Pareto front. To overcome these limitations, future research could consider to introduces innovative techniques that combine SA, symmetric perturbation, and chaos theory to improve the optimization process in multi-objective problems, promising the superior performance compared to eMOGOA.

## Data Availability

Upon request and subject to reasonable conditions, the corresponding author can provide the data, model, or code that underlie the findings of the study.

## Indices

*X*: Set of solutions
*Z*: Set of activities
*O*: Set of objectives
*i*: Index of the solution
*v*: Index of the solution dimension
*z*: Index of the activity
*o*: Index of the objective

## Parameters

*X*_*i*_: Position of the *i*th grasshopper
*S*_*i*_: Social interaction of the *i*th grasshopper
*G*_*i*_: Gravitational attraction exerted on the *i*th grasshopper
*A*_*i*_: Influence of wind and air circulation on the *i*th grasshopper
*l*: Current iteration
*L*: Maximum iteration
*n*: The number of solutions
*d*: The number of dimensions
*w*: The number of objectives
${x}_{i}^{v}$: The *v*th parameter of *i*th grasshopper
${x}_{T}^{v}$: The *v*th parameter of *i*th grasshopper selected through the TS process
*k*: Subset solutions selected through the TS process
*C*_*T*_: TS mechanism condition
*b*_*l*_: Lower bound of the1st dimension
*b*_*u*_: Upper bound of the 1st dimension
*b*_*l,v*_: Lower bound associated with the *v*th dimension
*b*_*u,v*_: Upper bound associated with the *v*th dimension
*TS*_*z*_: The start time of activity *z*th
*TF*_*z*_: The finish time of activity *z*th
*D*_*z*_: The duration of activity *z*th
*C*_*D*_: The sum of individual direct costs
*C*_*o*_: Financial expenses associated with operational budgets
*Q*_*ed*_: The electricity consumption associated with a specific activity
*Q*_*dd*_: The diesel consumption linked to the same activity
*Q*_*l*_: The usage of material *l* within the activity
*Q*_*e*_: The electricity consumption related to the transportation of material *l* for the activity
*Q*_*d*_: The diesel consumption pertaining to the transportation of material *l* for the activity
*F*_*e*_: The carbon emission factor (CEF) attributed to each unit of electricity consumption
*F*_*d*_: The CEF for each unit of diesel consumption
*F*_*l*_: The CEF for each unit of material *l* production

## Decision variables

${T}_{p}$: The project duration
${C}_{P}$: The overall project cost
*CE*_*p*_: The overall project cost
*avg*: Average value
*std*: Atandard deviation
*C*: C-metric
*DM*: Diversification measurement
*MID*: Mean ideal distance
*SP*: Spread
*HV*: Hyper-volume
*IGD*: Inverted generational distance

## References

[1] Koo C, Hong T, Kim S (2015). An integrated multi-objective optimization model for solving the construction time-cost trade-off problem. J. Civ. Eng. Manag..

[2] Tran D-H, Cheng M-Y, Prayogo D (2016). A novel multiple objective symbiotic organisms search (MOSOS) for time–cost–labor utilization tradeoff problem. Knowl.-Based Syst..

[3] Yan H (2010). Greenhouse gas emissions in building construction: A case study of One Peking in Hong Kong. Build. Environ..

[4] González MJ, Navarro JG (2006). Assessment of the decrease of CO_2_ emissions in the construction field through the selection of materials: Practical case study of three houses of low environmental impact. Build. Environ..

[5] Liu S, Tao R, Tam CM (2013). Optimizing cost and CO_2_ emission for construction projects using particle swarm optimization. Habitat Int..

[6] Khalili-Damghani K (2015). Solving multi-mode time–cost–quality trade-off problems under generalized precedence relations. Optim. Methods Softw..

[7] Sakellaropoulos S, Chassiakos A (2004). Project time–cost analysis under generalised precedence relations. Adv. Eng. Softw..

[8] Dong J (2023). Enhancing short-term forecasting of daily precipitation using numerical weather prediction bias correcting with XGBoost in different regions of China. Eng. Appl. Artif. Intell..

[9] Saremi, S. *et**al*. Grasshopper optimization algorithm: Theory, literature review, and application in hand posture estimation. In *Nature-Inspired**Optimizers:**Theories,**Literature**Reviews**and**Applications*. 107–122 (2020).

[10] Wu J (2017). Distributed trajectory optimization for multiple solar-powered UAVs target tracking in urban environment by Adaptive Grasshopper Optimization Algorithm. Aerosp. Sci. Technol..

[11] Barman M, Choudhury ND, Sutradhar S (2018). A regional hybrid GOA-SVM model based on similar day approach for short-term load forecasting in Assam, India. Energy.

[12] El-Fergany AA (2018). Electrical characterisation of proton exchange membrane fuel cells stack using grasshopper optimiser. IET Renew. Power Gener..

[13] Wang X (2023). Improved multi-objective grasshopper optimization algorithm and application in capacity configuration of urban rail hybrid energy storage systems. J. Energy Storage.

[14] Bukar AL (2020). A rule-based energy management scheme for long-term optimal capacity planning of grid-independent microgrid optimized by multi-objective grasshopper optimization algorithm. Energy Convers. Manag..

[15] Darvish Falehi A (2020). Optimal robust disturbance observer based sliding mode controller using multi-objective grasshopper optimization algorithm to enhance power system stability. J. Ambient Intell. Hum. Comput..

[16] Abualigah L, Diabat A (2020). A comprehensive survey of the Grasshopper optimization algorithm: Results, variants, and applications. Neural Comput. Appl..

[17] Aminbakhsh S, Sonmez R (2017). Pareto front particle swarm optimizer for discrete time-cost trade-off problem. J. Comput. Civ. Eng..

[18] Son, P. V. H. & Nguyen Dang, N. T. Optimizing time and cost simultaneously in projects with multi-verse optimizer. *Asian**J.**Civ.**Eng*. (2023).

[19] Parveen S, Saha SK (2012). GA based multi-objective time-cost optimization in a project with resources consideration. Int. J. Mod. Eng. Res. (IJMER).

[20] Son PVH, Nguyen Dang NT (2023). Solving large-scale discrete time–cost trade-off problem using hybrid multi-verse optimizer model. Sci. Rep..

[21] Gupta R, Trivedi MK (2022). AEHO: Apriori-based optimized model for building construction to time-cost tradeoff modeling. IEEE Access.

[22] Liu S, Meng X, Tam C (2015). Building information modeling based building design optimization for sustainability. Energy Build..

[23] Yi C-Y, Gwak H-S, Lee D-E (2017). Stochastic carbon emission estimation method for construction operation. J. Civ. Eng. Manag..

[24] He W (2021). Time, cost, and energy consumption analysis on construction optimization in high-rise buildings. J. Constr. Eng. Manag..

[25] Huynh V-H (2021). Multiple objective social group optimization for time–cost–quality–carbon dioxide in generalized construction projects. Int. J. Civ. Eng..

[26] Sharma K, Trivedi MK (2023). Discrete OBNSGA III method-based robust multi-objective scheduling model for civil construction projects. Asian J. Civ. Eng..

[27] Shehab M (2021). Enhanced a hybrid moth-flame optimization algorithm using new selection schemes. Eng. Comput..

[28] Manoharan P, Boggavarapu PKL (2021). Improved whale optimization based band selection for hyperspectral remote sensing image classification. Infrared Phys. Technol..

[29] Bakhshaei P, Askarzadeh A, Arababadi R (2021). Operation optimization of a grid-connected photovoltaic/pumped hydro storage considering demand response program by an improved crow search algorithm. J. Energy Storage.

[30] Zhenxing Z (2019). Antlion optimizer algorithm based on chaos search and its application. J. Syst. Eng. Electron..

[31] Al-Betar MA (2016). Tournament-based harmony search algorithm for non-convex economic load dispatch problem. Appl. Soft Comput..

[32] Tizhoosh, H.R. Opposition-based learning: A new scheme for machine intelligence. In *International**Conference**on**Computational**Intelligence**for**Modelling,**Control**and**Automation**and**International**Conference**on**Intelligent**Agents,**Web**Technologies**and**Internet**Commerce**(CIMCA-IAWTIC'06)* (IEEE, 2005).

[33] Wang H (2011). Enhancing particle swarm optimization using generalized opposition-based learning. Inf. Sci..

[34] Shaw B, Mukherjee V, Ghoshal S (2012). A novel opposition-based gravitational search algorithm for combined economic and emission dispatch problems of power systems. Int. J. Electric. Power Energy Syst..

[35] Wang H, Rahnamayan S, Wu Z (2013). Parallel differential evolution with self-adapting control parameters and generalized opposition-based learning for solving high-dimensional optimization problems. J. Parallel Distrib.Comput..

[36] Zhao F (2015). A shuffled complex evolution algorithm with opposition-based learning for a permutation flow shop scheduling problem. Int. J. Comput. Integr. Manuf..

[37] Luong D-L, Tran D-H, Nguyen PT (2021). Optimizing multi-mode time-cost-quality trade-off of construction project using opposition multiple objective difference evolution. Int. J. Construct. Manag..

[38] Pham VHS, Nguyen Dang NT, Nguyen VN (2023). Hybrid sine cosine algorithm with integrated roulette wheel selection and opposition-based learning for engineering optimization problems. Int. J. Comput. Intell. Syst..

[39] Pinto, H. *et**al*. A binary grasshopper algorithm applied to the knapsack problem. In *Artificial**Intelligence**and**Algorithms**in**Intelligent**Systems:**Proceedings**of**7th**Computer**Science**On-line**Conference**2018*. Vol. 2(7) (Springer, 2019).

[40] Crawford, B. *et**al*. A binary grasshopper optimisation algorithm applied to the set covering problem. In *Cybernetics**and**Algorithms**in**Intelligent**Systems:**Proceedings**of**7th**Computer**Science**On-line**Conference**2018*. Vol. 3(7) (Springer, 2019).

[41] Saxena, A. & Kumar, R. Chaotic variants of grasshopper optimization algorithm and their application to protein structure prediction. In *Applied**Nature-Inspired**Computing:**Algorithms**and**Case**Studies*. 151–175 (2020)

[42] Dwivedi S, Vardhan M, Tripathi S (2020). An effect of chaos grasshopper optimization algorithm for protection of network infrastructure. Comput. Netw..

[43] Mokeddem D (2021). Parameter extraction of solar photovoltaic models using enhanced levy flight based grasshopper optimization algorithm. J. Electric. Eng. Technol..

[44] Chhikara S, Kumar R (2020). MI-LFGOA: multi-island levy-flight based grasshopper optimization for spatial image steganalysis. Multimed. Tools Appl..

[45] Yue X, Zhang H, Yu H (2020). A hybrid grasshopper optimization algorithm with invasive weed for global optimization. IEEE Access.

[46] Guo S-S (2020). Improved grasshopper algorithm based on gravity search operator and pigeon colony landmark operator. IEEE Access.

[47] Bansal P (2020). A hybrid grasshopper and new cat swarm optimization algorithm for feature selection and optimization of multi-layer perceptron. Soft Comput..

[48] Sokolov, A. & Whitley, D. Unbiased tournament selection. In *Proceedings**of**the**7th**Annual**Conference**on**Genetic**and**Evolutionary**Computation* (2005).

[49] Zitzler E, Thiele L (1999). Multiobjective evolutionary algorithms: A comparative case study and the strength Pareto approach. IEEE Trans. Evolut. Comput..

[50] Wang L, Singh C (2009). Reserve-constrained multiarea environmental/economic dispatch based on particle swarm optimization with local search. Eng. Appl. Artif. Intell..

[51] Maghsoudlou H, Afshar-Nadjafi B, Niaki STA (2016). A multi-objective invasive weeds optimization algorithm for solving multi-skill multi-mode resource constrained project scheduling problem. Comput. Chem. Eng..

[52] Maghsoudlou H, Afshar-Nadjafi B, Niaki STA (2017). Multi-skilled project scheduling with level-dependent rework risk; Three multi-objective mechanisms based on cuckoo search. Appl. Soft Comput..

[53] Zitzler E (2003). Performance assessment of multiobjective optimizers: An analysis and review. IEEE Trans. Evolut. Comput..

[54] https://github.com/P-N-Suganthan/2020-Multimodal-Multi-Objective-Benchmark.

[55] Abdel-Basset M, Mohamed R, Abouhawwash M (2021). Balanced multi-objective optimization algorithm using improvement based reference points approach. Swarm Evolut. Comput..

[56] Lotfi R (2022). A robust time-cost-quality-energy-environment trade-off with resource-constrained in project management: A case study for a bridge construction project. J. Ind. Manag. Optim..

[57] Lotfi R (2022). Resource-constrained time–cost–quality–energy–environment tradeoff problem by considering blockchain technology, risk and robustness: A case study of healthcare project. Environ. Sci. Pollut. Res..

[58] Lotfi R (2023). Robust and resilience budget allocation for projects with a risk-averse approach: A case study in healthcare projects. Comput. Ind. Eng..

[59] Lotfi R (2017). Determination of start times and ordering plans for two-period projects with interdependent demand in project-oriented organizations: A case study on molding industry. J. Project Manag..

